# Porcine Models of Accelerated Coronary Atherosclerosis: Role of Diabetes Mellitus and Hypercholesterolemia

**DOI:** 10.1155/2013/761415

**Published:** 2013-06-13

**Authors:** Damir Hamamdzic, Robert L. Wilensky

**Affiliations:** Cardiovascular Division, Hospital of the University of Pennsylvania and Cardiovascular Institute, University of Pennsylvania, Philadelphia, PA 19104, USA

## Abstract

Animal models of atherosclerosis have proven to be an invaluable asset in understanding the pathogenesis of the disease. However, large animal models may be needed in order to assess novel therapeutic approaches to the treatment of atherosclerosis. Porcine models of coronary and peripheral atherosclerosis offer several advantages over rodent models, including similar anatomical size to humans, as well as genetic expression and development of high-risk atherosclerotic lesions which are similar to humans. Here we review the four models of porcine atherosclerosis, including the diabetic/hypercholesterolemic model, Rapacz-familial hypercholesterolemia pig, the (PCSK9) gain-of-function mutant pig model, and the Ossabaw miniature pig model of metabolic syndrome. All four models reliably represent features of human vascular disease.

## 1. Introduction

Atherosclerosis is a systemic disease affecting virtually all vascular beds. Primary and secondary prevention strategies, novel pharmaceutical treatment modalities, and early intervention have reduced mortality rates of coronary artery disease. However, cardiovascular atherosclerotic diseases such as acute coronary syndromes, stroke, and aortic disease continue to be the leading cause of death in developed countries and the incidence is rapidly increasing in developing countries. Macrovascular disease is the major cause of death with patients suffering from diabetes mellitus (DM), both type I and type II, having a 2- to 6-fold greater risk of developing atherosclerosis compared to nondiabetic patients with comparable risk factors [1]. Type II diabetic patients often exhibit increased low density lipoprotein (LDL) and decreased high density lipoprotein (HDL) cholesterol levels and hypertension (i.e., the metabolic syndrome), as well as altered platelet function. Often the diagnosis of type II DM is made at the time the patient presents with coronary artery disease. 

Animal models have proven invaluable in understanding the pathophysiology of atherosclerosis as well as developing and testing treatment strategies. Genetically modified murine models have led to an understanding of the mechanisms of disease and the role of signaling pathways and genetic factors which play a major role in disease initiation and development. However, mice are limited by their varying lipid profiles, lack of spontaneous coronary artery disease, and development of disease in vascular beds which are in variance with human disease [2, 3]. DM has only a small effect on development of atherosclerosis in mice [4], and their small size limits physiologic evaluation. Rabbits are limited because they do not naturally develop atherosclerosis and require a high cholesterol diet to induce atherosclerosis resulting in cholesterol levels which often exceed 1000 mg/dL, lesions which are largely foam cell rich, and, given size considerations, the need to perform vascular studies in the aortae and iliofemoral arteries rather than coronary arteries [5]. 

Such differences between rodents and humans have made it incumbent for additional models to be used to assess possible treatments, whether pharmacologic or device related. Porcine models of atherosclerosis have several advantages over small animal models. Pigs are closer phylogenetically to humans and present more similar anatomy, with a comparable size of the heart and vasculature. Like humans, pigs are omnivores and the vascular response to the increase in fat content of the diet is similar. Indeed, elderly pigs can spontaneously develop atherosclerosis [6]. In this review, we will focus on four porcine models with accelerated atherosclerosis: the induced diabetic/hypercholesterolemic (DM/HC) swine model, the LDL receptor knock-out model (or Rapacz pig), the recently described PCSK9 gain-of-function mutant cloned pig, and the Ossabaw metabolic syndrome model (Table 1).

## 2. Diabetic/Hypercholesterolemic Pig Model

In 2001, Gerrity et al. [7] first described superimposing DM on an HC diet in male Yorkshire domestic pigs. DM was induced by the intravenous injection of the pancreatic *β*-cell cytotoxin streptozotocin (STZ) which reduces the number of insulin producing *β*-cells, on average, by 90% at 2 weeks. Given the subsequent risk of hyperglycemic coma persistently elevated glucose levels, >350 mg/dL, are treated with administration of insulin [8]. An advantage of the model is that animals with persistently increased glucose levels grow at slower rates than normoglycemic animals. However, pigs with DM only do not develop atherosclerosis [7, 9, 10] and so in order to produce lesions DM pigs require a high fat/high cholesterol diet containing 5%–10% lard and 0.5%–1.0% cholesterol. The combination of DM and HC results in more severe lesions which are complex and humanoid in morphology [7–9].

**Figure 1:**
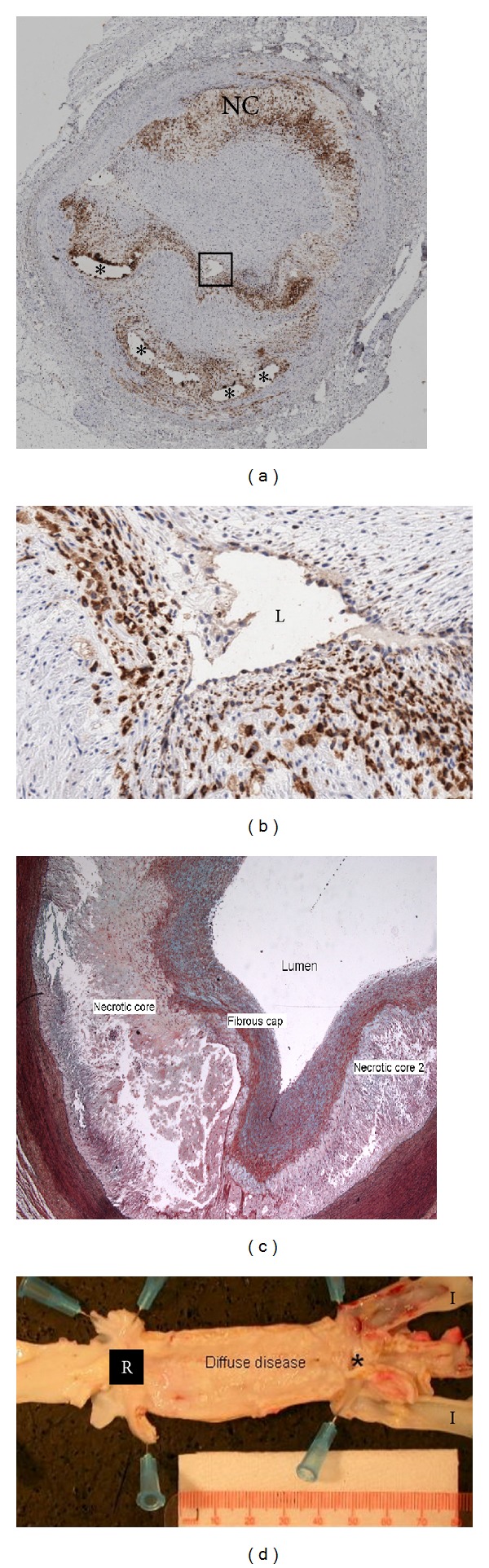
Examples of atherosclerotic lesions from DM/HC pigs. (a) Low power magnification of a coronary artery with a severe atherosclerotic lesion, obtained 6 months after DM/HC induction. Artery stained with Cathepsin S (brown cells) to denote the presence of inflammatory cells showing protease activity. Lumen is within the outlined box, *—calcification and NC-necrotic core. (b) High power view of outlined area in (a) with L denoting the arterial lumen. Cathepsin positive areas near the lumen indicate a thinned fibrous cap with increased inflammation. (c) Movat's staining of a high-risk coronary fibroatheroma showing two large necrotic cores covered by a fibrous cap. (d) Longitudinal view of the abdominal aorta demonstrating severe, diffuse atherosclerosis between the renal arteries (R) and the distal bifurcation (*) into the iliac arteries (I). The ostia of the iliac arteries are generally severely diseased in this model.

Development of atherosclerosis in DM/HC pigs is variable in presence, severity, morphology, and vascular location of the lesions. Lesions develop in all animals in the distal, infrarenal abdominal aorta while less severe lesions are observed in thoracic aortae. Coronary arteries demonstrate variable lesion development with some animals developing severe lesions, occasionally resulting in sudden cardiac death; some animals develop single severe lesion while others develop nonhemodynamically significant lesions or no lesion at all. The most advanced lesions are observed in the proximal segment of the coronary arteries. Only a minority of carotid arteries develop lesions. Early lesions in the affected vascular beds are observed 3 months after induction [7, 11] and are characterized as either intimal thickening or xanthomas consisting of lipid deposits, foam cells, and inflammation (mainly the early recruitment of macrophages). By 3 months 61% of coronary arteries, 20% of carotids, and 89% of thoracic aortae demonstrate a lesion increasing to 96%, 31%, and 100% at 6 months, respectively. By 6 months atherosclerotic lesions in coronary arteries and abdominal aorta of DM/HC pigs progress into complex human-like atherosclerotic plaques characterized by lipid pools and lipid-rich necrotic cores, thinning of the fibrous cap, increased macrophage infiltration, calcification, and destruction of the media (Figure 1). By 9 months high-grade complex lesions consisting of smooth muscle cells, extracellular matrix, increased calcifications and necrotic cores are observed. Abdominal aortic lesions demonstrate large necrotic cores and Monckeberg's sclerosis. At 9 months all coronary arteries, one-third of carotids, and all thoracic aortae have lesion development [11]. Advanced lesions such as fibroatheromas are observed in 6% of coronary arteries at 3 months, 17% at 6 months, and 39% at 9 months and fibrocalcific plaques are noted in 0%, 2%, and 22% of lesions at the 3 time points [11]. Morphologically advanced coronary artery lesions mimic human diabetic lesions [12, 13].

Akt is a central signaling node important for inflammation and involved in vascular cell growth, proliferation, differentiation, apoptosis, and angiogenesis. Reduced Akt activation results in loss of vascular protection contributing to increased vascular inflammation and disease progression. A possible mechanism by which the combination of DM and HC produces complex lesions is through aberrant, reduced p-Akt activity. Hamamdzic et al. [10] showed that compared to control, DM only, and HC only coronary arteries, DM/HC coronary arteries demonstrate greater attenuated Akt activity resulting in decreased phosphorylation of glycogen synthetase kinase-3*β* (GSK-3*β*). This in turn was associated with increased cellular proliferation and apoptosis as well as activation of nuclear factor-kappa B (NF-*κ*B) resulting in increased complexity of the atherosclerotic lesions. Aberrant Akt signaling also correlated with increase vasa vasorum neovasculogenesis.

Evaluation of 59 genes obtained from DM/HC coronary, carotid, and thoracic aortic arteries, 1, 3, and 6 months after DM/HC induction, showed that genes involved in cholesterol metabolism and insulin pathways were most markedly upregulated in coronary arteries; more so than in thoracic aortae and carotid arteries. At 1 month increased gene expression of insulin related pathways, such as adiponectin, leptin, PPAR*γ*, and preproadipsin, was noted in coronary arteries [11] and early increased expression of inflammatory genes was observed only in coronary arteries [11], while at 6 months increased expression of multiple genes, including those associated with monocyte chemotaxis, progression of macrophages to foam cells, cell growth, and maintenance was observed in coronary arteries. Genes implicated in plaque instability were upregulated only in the coronary arteries at the 6-month time point.

Using intravascular ultrasound (IVUS) imaging of coronary arteries in DM/HC pigs, Koskinas et al. [14] demonstrated that low endothelial sheer stress (ESS) was an independent predictor of the development and progression of atherosclerosis and importantly with the development of high-risk coronary lesions. Furthermore, plaques with excessive remodeling in the areas of very low ESS were associated with additional plaque growth resulting in development of high-risk atherosclerotic lesions with increased expression of collagenases in the fibrous cap [14, 15]. Similar results demonstrating the relationship between low shear stress and plaque progression have been obtained in patients following an acute coronary syndrome thereby indicating the relevance of the DM/HC model for investigating lesion development [16]. 

Using Near Infrared Spectroscopy (NIRS) and IVUS imaging, obtained at 3, 6, and 9 months after DM/HC induction, Patel et al. [17] demonstrated that the combination of NIRS and IVUS could detect the presence of a fibroatheroma and importantly predict the future development of fibroatheromas. The early and persistent accumulation of lipid within the arterial wall of coronary arteries led to the subsequent development of fibroatheromas, often demonstrating increased inflammation in the plaque and within the fibrous cap as well as thinned fibrous caps; markers of high-risk lesions. 

The vascular effects of both drug-eluting and bare-metal stenting in DM/HC porcine coronary arteries were examined and compared to implantation in nonatherosclerotic porcine arteries [18]. Increased neointimal accumulation and vascular inflammation following both bare metal and paclitaxel eluting stent implantation were observed in DM/HC swine, and use of the DM/HC model allowed for greater discrimination of the vascular effects of bare metal and drug eluting stents compared to nonatherosclerotic porcine model. DM/HC pigs also demonstrated increased platelet aggregation mimicking the findings in diabetic patients. In addition, the findings of increased vascular inflammation and delayed reendothelization in DM/HC porcine arteries as well as increased platelet reactivity lent insight into the possible mechanisms of subacute and late stent thrombosis, a potentially deadly untoward effect of drug eluting stent implantation. 

Since vascular inflammation plays an important role in development of atherosclerosis and specifically high-risk lesions in human and DM/HC porcine coronary arteries, it was hypothesized that selective inhibition of lipoprotein associated phospholipase A_2_ (Lp-PLA_2_) could reduce lesion development in this model [8]. Darapladib, a selective inhibitor of Lp-PLA_2_, inhibited plasma and vascular Lp-PLA_2_ activity thereby reducing levels of lysophosphatidylcholine and oxidized nonesterified fatty acids, inflammatory mediators which are released by the action of Lp-PLA_2_ on intramural oxidized fatty acids. Analysis of coronary gene expression showed that darapladib exhibited a general anti-inflammatory action by reducing the expression of the genes associated with macrophage and T lymphocyte functioning, independent of an effect on blood cholesterol levels. Morphologically, selective Lp-PLA_2_ inhibition resulted in decreased plaque and necrotic core area and medial destruction. As a consequence selective Lp-PLA_2_ inhibition resulted in fewer lesions with an unstable phenotype. This study corroborated the results of the IBIS 2 study [19] in which the treatment reduced necrotic core area in human coronary artery lesions, as determined by VH-intravascular ultrasound over a year in patients with coronary artery disease and led to two large multicenter, international Phase III clinical trials examining the use of darapladib to prevent primary and secondary cardiovascular events. 

## 3. LDLR−/− Pig Model of Familial Hypercholesterolemia

An unique strain of pigs exhibiting elevated LDL levels and spontaneous atherosclerosis, due to a mutation in genes coding for apolipoproteins and LDL receptor, akin to familial hypercholesterolemia, has been described and has become known a Rapacz-FH pigs [20, 21]. When fed low fat/low cholesterol diet, total blood cholesterol values in these animals ranged from 234 to 464 mg/dL, compared to 96 to 113 mg/dL in the control pigs [22]. Blood levels of triglycerides were 48 ± 10.8 mg/dL in FH animals, compared to 29 ± 5.7 mg/dL in control group, while apoB levels in FH group were 152 ± 32.5 mg/dL, compared to 48 ± 5.7 mg/dL in control group. 

**Figure 2:**
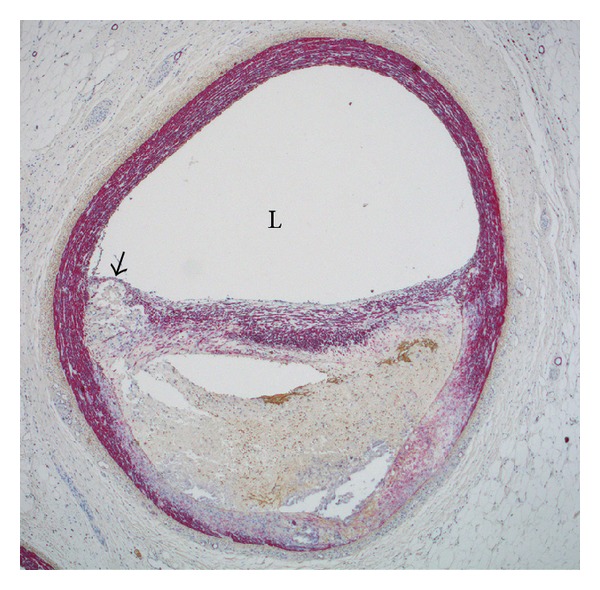
Photomicrograph of a lesion from an eccentric 13-month-old Rapacz-FH minipig coronary artery. Arrow denotes a thin fibrous cap covering a foam cell rich area with necrotic cores (empty areas within the lesion). The pig was fed a mixed high/fat, low/fat diet. Photomicrograph kindly provided by Dr. Erling Falk.

Immature atherosclerotic lesions were identified at 12 months of age and were characterized by the presence of macrophage derived foam cells and smooth muscle cell proliferation in the coronary arteries, iliofemoral arteries, and aorta. By 18 months of age 60% of animals exhibited atheromas. Lesions were most prominent in the coronary arteries (Figure 2), but peripheral lesions were also observed in thoracic aortae and at the aortoiliac bifurcation. By 24 months, fully developed atherosclerotic lesions were seen in coronary arteries, characterized by necrotic cores with thinned fibrous caps, cholesterol deposits, calcification and neovascularization. By 39–54 months advanced lesions were present in the coronary, iliac, and femoral arteries, characterized by extensive calcification, necrotic cores, fibrous caps, and inflammatory cells. Neovascularization and intraplaque hemorrhage were also present indicating a high risk of subsequent plaque rupture [20]. The pathogenesis of atherosclerosis in this model mimics the progression of human disease and is potentially very useful for testing novel treatment and imaging modalities. However, major limitations are the slow progression of the disease (12–36 months) and the size of the animals, often exceeding 200 kg. More rapid lesion development results from administration of a high cholesterol diet and balloon induced focal coronary injury [23]. 

Rapacz FH pigs develop peripheral arterial atherosclerosis in femoral but rarely in brachial arteries. Bahls et al. [24] investigated gene expression profiling to investigate the differences between these two vascular beds in young, disease-free pigs to determine how the expression of pro-atherogenic genes contributes to the heterogeneous distribution of atherosclerosis development. These investigators showed that the differences in gene expression between healthy brachial (atheroprotected) and femoral (atherosusceptible) arteries exist prior to the onset of atherosclerotic disease. The gene expression profiles between the two arteries were not similar in that only 430 of 15,552 exhibited genes significantly demonstrated different levels of expression. Ribosomal structural genes essential for the formation of the large 60S ribosomal subunit which controls protein production and the small 40S ribosomal subunit which programs protein synthesis, binds mRNA, and mediates the interactions between mRNA codons and transfer RNA anticodons were differentially expressed. Even though distinct functions during translation of these proteins have been described, the possible consequence of an overexpression of either the large or the small ribosomal subunits remains unclear.

Rapacz FH swine have been used to investigate whether angioplasty with the zotarolimus coated balloon (ZCB) would inhibit neointimal hyperplasia in balloon injured superficial femoral arteries [25]. Zotarolimus, a molecular target of rapamycin (m-TOR) inhibitor, has been shown to inhibit cell-cycle progression and is used clinically, from a stent platform, to reduce restenosis. Pharmacokinetic studies after balloon denudation in superficial femoral artery segments, followed by balloon angioplasty, revealed that zotarolimus was detected in arterial tissue up to 28 days after ZCB inflation and was associated with a reduction in neointimal formation compared with controls. There was no evidence of delayed arterial healing or vascular toxicity in ZCB animals.

Given the unwieldy size and weight of full grown Rapacz pigs, a smaller, more manageable FH pig has been developed by crossing the Rapacz farm pigs with smaller Chinese Meishan pigs and then crossing the offspring with a smaller minipig [26]. Atherosclerosis in these animals was induced by high fat/high cholesterol diet and accelerated by coronary artery balloon injury. Following cholesterol feeding (resulting in a 4-fold increase in total cholesterol levels) 22 high-risk atherosclerotic lesions containing a large necrotic core and thin fibrous cap were observed in 18 arteries which developed either spontaneously and following balloon injury. Of interest, there was a high incidence of intraplaque hemorrhage and neovascularization as well as expansive remodeling in the lesions, signs of rapidly progressing, unstable lesions. The lesions are similar morphologically to the DM/HC coronary lesions despite the absence of DM.

## 4. (PCSK9) Gain-of-Function Mutant Pig

Recently, a new Yucatan minipig model of familial hypercholesterolemia was created by DNA transposition of human D374Y-proprotein convertase subtilisin/kexin type 9 (PCSK9) gain-of-function mutant [27]. The D374Y-PSSK9 gain-of-function mutation in the proprotein convertase subtilisin/kexin type 9 (PCSK9) gene causes severe autosomal dominant hypercholesterolemia and early development of atherosclerosis in humans. Clinical studies have shown that the subcutaneous administration of a monoclonal antibody to PCSK9 reduced LDL levels in a dose-dependent manner in up to 61% in patients with familial or nonfamilial hypercholesterolemia [28].

**Figure 3:**
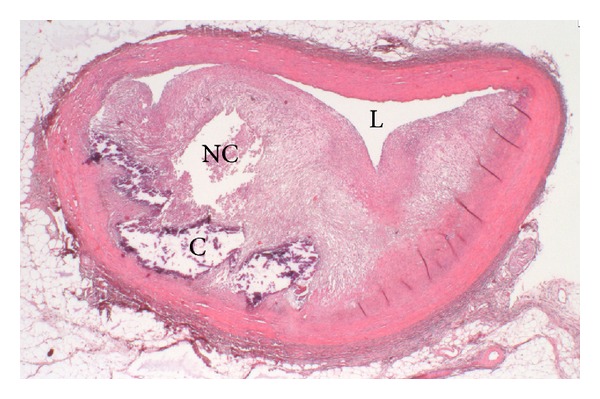
Photomicrograph of a high-grade coronary atherosclerotic lesion obtained from a 13-month PCSK9 gain-of-function mutant pig fed a high cholesterol diet. C: calcifications; L: lumen; NC: necrotic core; Photomicrograph kindly provided by Dr. Erling Falk.

Al-Mashhadi et al. [27] showed that Yucatan minipigs expressing human, liver specific D374Y-PCSK9 exhibited reduced LDL receptor levels, resulting in impaired LDL clearance and increased plasma LDL levels. The administration of a high fat/high cholesterol diet for 46 weeks led to greatly increased cholesterol (approximately 772 mg/dL or 20 mM) and LDL (425 mg·dL or 11 mM) levels. At approximately 1 year of age complex progressive human-like atherosclerotic lesions in the aortae and iliofemoral and left anterior descending coronary arteries were observed. Mean aortic surface area covered by atherosclerosis increased by 2.1-fold in transgenic pigs compared with wild type. In the abdominal aorta lesions characterized as intimal thickening or fibroatheromas were present in all transgenic males, while only 28% of control wild-type (w/t) animals exhibited similar type lesions. In the iliofemoral arteries there was a 7.6-median-fold increase in atherosclerosis, while the left anterior descending coronary arteries demonstrated a 1.8-fold increase compared to wild-type males. All left anterior descending coronary arteries demonstrated at least one advanced lesion compared to only 28% in the wild-type pigs. An example of the lesions is noted in Figure 3. [18^F^] Fluorodeoxyglucose (FDG) Positron emission tomography (PET) detected inflammatory activity in the aorta. As such, the (PCSK9) gain-of-function mutant pig is a potentially important large-animal translational research model. 

## 5. Metabolic Disease Pig Model (Ossabaw Pig)

The DM/HC porcine model is a type I diabetic model while the majority of DM patients with diabetes suffer from type II diabetes and metabolic syndrome (MetSyn). The lack of a viable and reproducible, human-like animal model of MetSyn with type II DM has hampered the advances in this area of research. However, the Ossabaw strain of pigs offers promise in lending itself to advancing the understanding of the pathophysiology of MetSyn as well as the testing of novel treatment strategies. 

In the early 16th century Spanish explorers left a small herd of pigs on Ossabaw Island located off the coast of Georgia (USA). As a result of their geographic isolation the pigs adapted to conditions on the island, characterized by extreme seasonal tides, by evolving a smaller phenotype (insular dwarfism) while adapting to the food cycle which provided minimal amount of nutrition during the spring season. The development of a unique, thrifty genotype afforded these pigs the ability to store large amounts of fat which was used for survival during periods of famine but also increased the propensity for obesity. As a result this strain has developed low-grade, non-insulin-dependent diabetes when fed an atherogenic diet of cholesterol and fat derived from hydrogenated soybean oil and fructose. In addition to the increased risk of developing diabetes, Ossabaw swine fed this type of diet nearly double their percentage body fat in only 9 weeks showing signs of MetSyn including obesity, insulin resistance, hypertension, and dyslipidemia with an increase in the LDL/HDL cholesterol ratio and hypertriglyceridemia [29]. Female obese pigs compared with lean pigs demonstrated a 2-fold increase in insulin × glucose concentrations and an over 4-fold increase in total cholesterol (156.6 ± 13.4 mg/dL versus 479.0 ± 46.7 mg/dL) with the greatest increase in LDL levels (104.2 ± 13.2 mg/dL versus 430.8 ± 48.0 mg/dL). The mean blood pressure was increased from 92 ± 9 mmHg to 113 ± 8 mmHg [30].

**Figure 4:**
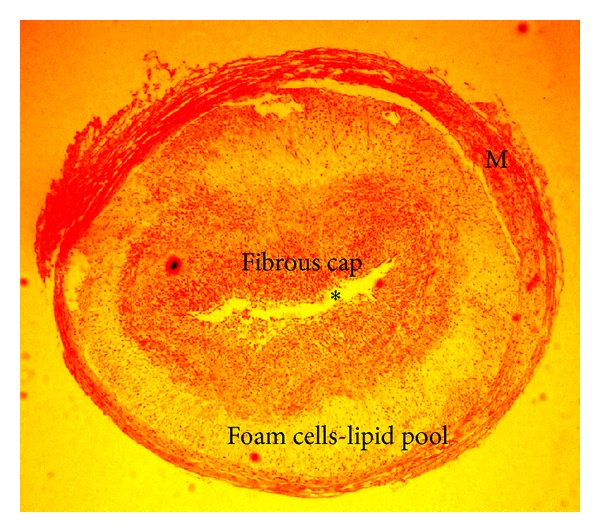
Photomicrograph of a coronary lesion obtained from an Ossabaw pig on a high fat diet. Lumen denoted by *, and M indicates medial layer. Kindly provided by Drs. Michael Sturek and Mouhamad Alloosh, Department of Cellular and Integrative Physiology, Indiana University School of Medicine.

Mild, early coronary atherosclerosis, characterized as intimal thickening, was also observed [28]. Neeb et al. compared the features of MetSyn, coronary artery disease, and stent induced restenosis in Ossabow pigs to a Yucatan miniature pigs, both fed either a control or high fat diet with calorie-matching for 40 weeks [31]. An example is shown in Figure 4. Ossabaw pigs with MetSyn had more extensive and diffuse native CAD, defined as wall coverageas well as a 2.5-fold greater accumulation of neointimal hyperplasia, 3 weeks following stent implantation. There was little difference in the severity of the in-stent restenotic lesions between those Ossabaw pigs on a control diet and those on a high fat diet. Of interest was that Ossabaw coronary arteries were less fibrous and more cellular, compared to Yucatan coronary arteries, indicating a potentially higher risk for the plaque instability and subsequent thrombosis. Increased platelet activity has been shown in the MetSyn Ossabaw pigs compared to lean non-MetSyn pigs [32], mimicking that observed in patients with diabetes.

## 6. Conclusion

Large animal models of human-like atherosclerosis are of increasing importance in preclinical studies evaluating novel treatments and imaging techniques and further elucidating the pathophysiology of cardiovascular diseases. We have described here the four viable pig models that hold promise in translating preclinical results to human treatment. The major limitations for the pig studies remain the size of the animal (relevant to housing requirements), length of the studies, and cost. While each of these models has specific uses as well as potential limitations, continuing elaboration and optimization of these models is ongoing.

## Figures and Tables

**Table 1:** Relative advantages and disadvantages of the 4 atherosclerotic porcine models.

Model	Advantages	Limitations
Diabetes/ hypercholesterolemic	Well characterized. Reproducible human-like atherosclerosis. Results obtained from diagnostic and pharmacologic treatment studies corroborate data obtained in humans. Complex lesions detectable as early as 6 months after induction.	Type I diabetic model. Variable development of atherosclerosis.

Rapacz familial hypercholesterolemic	Well characterized Models a known human disease state.	Long induction period for atherosclerosis. Large size of animals (although genetic modified mini-pig has been developed). Expensive.

PCSK9 gain of function	Small size. Models a known human disease state. Reproducible lesions.	Limited commercial availability. Relatively long-term induction period (12 months).

Ossabaw	Only model of metabolic syndrome induced atherosclerosis. Small size. Relatively short induction period after a high fat diet regimen.	Limited commercial availability.
